# When Surgical Intervention Becomes a Risk: Severe Drug‐Induced Gingival Overgrowth and Clinical Decision‐Making in Alpers–Huttenlocher Syndrome

**DOI:** 10.1111/scd.70241

**Published:** 2026-08-24

**Authors:** Marcus Vinícius Bueno, Renata Zoraida Rizental Delgado, Karem Ortega, Juliana Bertoldi Franco

**Affiliations:** ^1^ Dentistry Division – Central Institute – Hospital Das Clínicas, School of Medicine University of São Paulo São Paulo São Paulo Brazil; ^2^ Department of Stomatology, School of Dentistry University of São Paulo São Paulo São Paulo Brazil; ^3^ Department of Dentistry, Children and Adolescents Institute Clinical Hospital of Medical School of the University of São Paulo São Paulo São Paulo Brazil; ^4^ School of Dentistry State University of Northern Paraná Jacarezinho Paraná Brazil; ^5^ Department of Dentistry Clinics Hospital of the State University of Campinas (UNICAMP) Campinas São Paulo Brazil

**Keywords:** Alpers Huttenlocher syndrome, gingival hypertrophy, gingivoplasty, intensive care unit, oral care

## Abstract

**Introduction:**

Alpers–Huttenlocher syndrome (AHS) is an ultra‐rare, autosomal recessive mitochondrial encephalopathy caused by pathogenic *POLG* variants, characterized by refractory epilepsy, hepatic dysfunction, and progressive neurodegeneration. Drug‐induced gingival overgrowth (DIGO) is a recognized complication of anticonvulsant and immunosuppressive therapy, particularly in medically complex patients. This report describes severe DIGO in a child with genetically confirmed AHS and explores the implications for multidisciplinary dental management.

**Methods:**

A CARE‐compliant case analysis was conducted in a five‐year‐old girl with AHS, status post living‐donor liver transplantation, chronically hospitalized in a long‐term intensive care unit. Clinical findings, pharmacologic exposures, multidisciplinary decision‐making, and conservative oral management strategies were systematically documented.

**Results:**

The patient, ventilator‐dependent and gastrostomy‐fed, had prolonged exposure to phenytoin, sodium valproate, topiramate, and tacrolimus without prior dental surveillance. Examination revealed severe fibrotic maxillary gingival overgrowth, delayed tooth eruption, and functional compromise. Given her profound systemic fragility, gingivoplasty under general anesthesia was contraindicated. A structured conservative protocol including 0.12% chlorhexidine, mucosal care, and continuous dental follow‐up was implemented to reduce inflammatory and infectious burden.

**Conclusion:**

This case underscores the necessity of integrating dental care into the multidisciplinary management of rare mitochondrial disorders and represents, to our knowledge, the first reported association between AHS and severe DIGO.

## 1 Introduction

Alpers–Huttenlocher syndrome (AHS) is an ultra‐rare, autosomal recessive mitochondrial disorder within the spectrum of *POLG*‐related diseases and represents one of the most severe pediatric phenotypes associated with *POLG* mutations, which themselves account for a significant proportion of inherited mitochondrial DNA maintenance disorders [1, 2]. Population‐based estimates suggest an incidence of approximately 1 per 100,000–200,000 live births, although precise epidemiological data remain limited due to underdiagnosis and phenotypic heterogeneity. The condition demonstrates a bimodal age distribution, with the classical early‐onset form presenting between 2 and 4 years of age and a less common juvenile or young adult form emerging during adolescence. No consistent sex predilection has been identified [1, 2].

The prognosis of AHS remains extremely poor, with historical cohorts demonstrating that most affected children die within a few years after symptom onset, predominantly as a consequence of progressive hepatic failure, refractory status epilepticus, or severe neurodegenerative encephalopathy [1, 2, 3]. Although advances in molecular diagnostics and next‐generation sequencing have substantially improved early recognition and diagnostic accuracy, the true global burden of the disease is likely still underestimated, particularly in low‐ and middle‐income regions where access to comprehensive genetic testing and systematic screening for mitochondrial disorders remains limited [1, 2, 3].

Recent advances in the understanding of POLG‐related disorders have shown that DNA polymerase gamma dysfunction causes not only mitochondrial DNA depletion and deletions, but also profound bioenergetic instability and activation of mitochondrial stress pathways. These alterations predominantly affect tissues with high metabolic demand, especially the central nervous system and liver, contributing to the severe neurodegenerative and hepatic manifestations of Alpers–Huttenlocher syndrome [4, 5].

Current evidence suggests that these disorders represent states of dynamic mitochondrial instability, in which metabolic stressors, infections, and chronic polypharmacy may aggravate disease severity. Investigational therapies such as deoxynucleoside supplementation have been explored to improve mitochondrial DNA replication and cellular energetics [6].

Drug‐induced gingival overgrowth (DIGO) represents a clinically significant and mechanistically complex adverse effect primarily associated with anticonvulsants, calcineurin inhibitors, and calcium channel blockers. Classic agents such as phenytoin, cyclosporine, tacrolimus, nifedipine, and amlodipine have been consistently implicated. However, the severity and prevalence vary according to drug class, dosage, duration of exposure, and host susceptibility [7, 8].

The pathobiology of DIGO extends beyond simple drug toxicity; it reflects a dysregulated interplay between pharmacologic modulation of intracellular calcium flux, altered fibroblast phenotype, reduced collagenase activity, and enhanced extracellular matrix deposition, particularly type I collagen [9, 10]. Genetic predisposition, gingival fibroblast heterogeneity, and local inflammatory burden driven by dental biofilm further modulate clinical expression. Importantly, chronic inflammation acts synergistically with systemic pharmacologic effects to amplify fibroproliferative responses, underscoring the bidirectional relationship between systemic therapy and oral health [9, 10, 11]. Contemporary evidence suggests that early identification, rigorous plaque control, and multidisciplinary collaboration are critical to mitigating disease progression, particularly in medically complex patients exposed to long‐term polypharmacy [10, 11].

It is well established in the literature that dental biofilm plays a fundamental role in the development and progression of DIGO [9, 10], while oral health status reflects the patient's overall systemic health [12, 13]. Regular dental consultations should therefore be recommended to control dental biofilm formation as a preventive strategy against GO and to provide appropriate guidance on maintaining oral health [9, 10, 11].

The objective of this study was to report a clinical case of DIGO in a patient diagnosed with AHS. To the best of our knowledge, this is the first clinical case report describing an association between AHS and drug‐induced alterations in gingival tissue.

## 2 Case Report

A five‐year‐old female with genetically confirmed AHS, status post living‐donor liver transplantation at two years of age, was evaluated during prolonged hospitalization in a long‐term intensive care unit. Her clinical trajectory had been marked by refractory epilepsy, progressive neurological decline, and chronic critical illness. At the time of dental consultation, she was ventilator‐dependent by tracheostomy, unconscious, and non‐responsive to external stimuli, receiving exclusive enteral nutrition through gastrostomy. She remained on continuous multidrug therapy, including phenytoin, topiramate, sodium valproate, and tacrolimus. Notably, before referral to our tertiary care center, the patient had remained without any form of dental surveillance or intervention for approximately four years, underscoring a prolonged period of unmonitored oral disease progression. Written informed consent for publication of the patient's clinical data and accompanying images was obtained from the patient's legal guardian prior to manuscript preparation.

**FIGURE 1:**
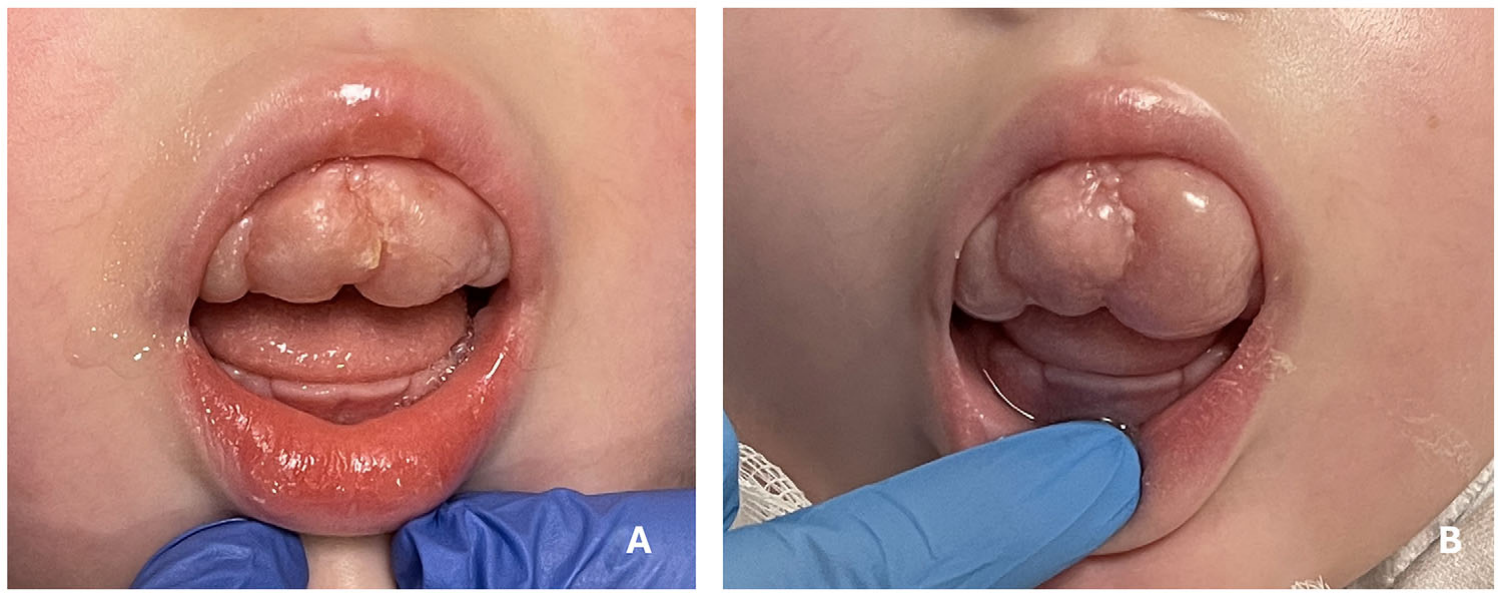
(A) Intraoral findings showing severe maxillary DIGO characterized by dense fibrotic enlargement, protrusion with persistent oral cavity exposure, and complete absence of erupted primary dentition secondary to mechanical obstruction by hypertrophic gingival tissue; (B) Clinical appearance at the 1‐year follow‐up demonstrating progressive enlargement of the diffuse DIGO.

Comprehensive intraoral examination revealed severe and disproportionate maxillary gingival overgrowth, characterized by dense, fibrotic, lobulated tissue extending beyond the alveolar ridges with persistent protrusion and continuous exposure of the oral cavity. There was a complete absence of erupted primary dentition in the maxillary arch, with clinical evidence of delayed tooth eruption secondary to mechanical obstruction by the hypertrophic gingiva. The marked alteration of oral anatomy resulted in significant esthetic deformity, impaired lip competence, increased mucosal dehydration, and potential compromise of local tissue integrity. The gingival phenotype was predominantly fibrotic, with limited overt inflammatory signs, consistent with advanced DIGO in the context of long‐term anticonvulsant and immunosuppressive therapy compounded by the absence of routine oral care (Figure 1).

Given the extent of tissue proliferation and the patient's profound systemic fragility, the case was deliberated in depth with the multidisciplinary team, including intensivists, neurologists, and transplant specialists. This discussion was pivotal in guiding clinical decision‐making, particularly in balancing surgical benefits against infectious risk and anticipated challenges in postoperative healing. The potential reduction or modification of anticonvulsant and immunosuppressive therapy was carefully considered; however, given the critical need for seizure control and graft preservation, tapering phenytoin, sodium valproate, or tacrolimus was deemed clinically inappropriate.

A structured, conservative oral care protocol was therefore instituted, consisting of twice‐daily application of 0.12% chlorhexidine, meticulous mucosal cleansing, and continuous hydration with a water‐based lubricant to reduce the inflammatory burden and mitigate infectious risk. Although surgical gingivectomy under general anesthesia was contemplated as definitive management, operative intervention was deferred pending systemic stabilization and comprehensive perioperative risk assessment. The patient remains under weekly follow‐up by the dental team, without clinical complications or progression of the DIGO.

This case exemplifies the essential role of multidisciplinary collaboration in the care of medically complex patients and highlights the necessity of integrating dental expertise into the broader framework of critical and transplant care.

## 3 Discussion

AHS is an ultrarare and severe POLG‐related mitochondrial encephalopathy, with estimated prevalence of approximately 1 in 100,000 children and typical onset during early childhood [2, 3]. The disorder is characterized by marked phenotypic heterogeneity, progressive neurodegeneration, refractory epilepsy, and hepatic dysfunction, often creating substantial diagnostic challenges, particularly when neurological manifestations precede liver involvement. Current evidence indicates that AHS should be interpreted as a dynamic mitochondrial disorder, in which metabolic stressors, infections, and cumulative pharmacologic exposure may accelerate clinical deterioration [1, 2, 3]. Early molecular diagnosis, especially during infancy, may therefore reflect either increased genomic surveillance or a more aggressive disease phenotype.

At the molecular level, POLG dysfunction impairs mitochondrial DNA replication, leading to mtDNA depletion, bioenergetic failure, and activation of mitochondrial stress pathways, predominantly affecting high‐energy tissues such as the brain and liver [4, 5]. Experimental evidence further suggests that inflammatory and metabolic stressors intensify mitochondrial instability, contributing to rapid neurological and systemic decline [4, 5]. Emerging therapeutic approaches, including deoxynucleoside supplementation, have shown potential to partially restore mitochondrial function in experimental models, representing a promising shift toward mechanism‐based therapies in POLG‐related disorders [4, 5, 6].

Clinically, AHS is characterized by refractory epilepsy as its earliest and most defining manifestation, reflecting impaired oxidative phosphorylation, ATP depletion, and progressive neurodegeneration [3]. Additional features, including blindness, ataxia, spastic paraparesis, and cognitive decline, underscore the multisystemic nature of mitochondrial compromise [2]. The patient described exhibited a severe phenotype with intractable seizures progressing to hepatic failure, consistent with advanced mitochondrial dysfunction. While electroencephalography is recommended as a diagnostic adjunct [2], it was not performed due to pathognomonic clinical and genetic findings.

Liver transplantation remains controversial, considered contraindicated by some in advanced disease [14], yet associated with potential metabolic stabilization in selected cases, highlighting the necessity of individualized clinical judgment. Despite emerging therapeutic advances, management remains largely supportive and palliative, and survival beyond four years from symptom onset remains uncommon, emphasizing the urgent need for early, multidisciplinary intervention [2, 3]. The relentlessly progressive trajectory of AHS mandates structured, proactive, and truly multidisciplinary management. Advanced neurological decline frequently culminates in severe dysphagia and loss of protective airway reflexes, rendering gastrostomy placement necessary for long‐term nutritional support, as observed in the present patient.

In this context of chronic critical illness, each supportive intervention becomes integral to survival and quality of life. Equally important, and often underestimated, is the incorporation of dental care into the multidisciplinary framework. As demonstrated by Franco et al. (2014) [12] and Bassan et al. (2018) [13], structured oral care protocols in intensive care settings play a pivotal role in reducing biofilm accumulation and preventing secondary complications, including ventilator‐associated pneumonia (VAP).

Gingival alterations in critically ill patients are frequently underrecognized; however, unchecked oral inflammatory burden may amplify systemic stress through transient bacteremia, sustained cytokine release, and heightened immune activation [1, 7, 8, 9, 10, 11, 12, 13]. In medically fragile individuals with mitochondrial dysfunction, even localized inflammatory processes may carry disproportionate systemic consequences. The formal request for dental evaluation in this case exemplifies coordinated, multidisciplinary care. It reinforces the principle that oral health is not ancillary but rather a determinant of systemic stability in complex pediatric patients. By integrating dental expertise into critical care management, the team acknowledged the oral cavity as a potential source of modifiable inflammatory load, thereby aligning local intervention with global clinical objectives.

DIGO is a well‐established adverse effect associated with anticonvulsants, calcineurin inhibitors, and calcium channel blockers, with a pathobiology that extends far beyond simple drug accumulation in gingival tissues. The underlying mechanism involves dysregulated gingival fibroblast metabolism, enhanced extracellular matrix synthesis, particularly collagen type I, reduced collagenase activity, and altered cytokine signaling in genetically predisposed individuals [7]. At the molecular level, agents such as tacrolimus and certain anticonvulsants have been implicated in promoting fibroblast proliferation through calcium‐dependent intracellular pathways and upregulation of transforming growth factor‐beta (TGF‐β), thereby favoring a profibrotic microenvironment [7, 8, 9, 10, 11].

In the context of mitochondrial dysfunction, as seen in POLG‐related disease, impaired cellular bioenergetics may further compromise tissue homeostasis, amplifying fibroproliferative and inflammatory responses [4, 7, 8, 9, 10, 11]. Mitochondrial instability may hinder adequate regulation of oxidative stress and extracellular matrix turnover, creating a biologically permissive setting for exaggerated gingival enlargement. In the present patient, the chronic administration of phenytoin, topiramate, sodium valproate, and tacrolimus therapy, combined with prolonged ICU dependency and severely limited mechanical plaque control, likely generated a synergistic pathogenic milieu conducive to exuberant and disfiguring gingival overgrowth.

Although Tungare and Paranjpe (2021) [7] report a higher prevalence of DIGO in males and a predilection for anterior gingival regions, this case underscores that severe and functionally compromising manifestations may arise irrespective of sex when multiple pharmacologic exposures converge with a persistent local inflammatory burden. The clinical severity observed here reflects not merely medication effect, but the convergence of polypharmacy, mitochondrial vulnerability, and sustained oral biofilm accumulation within a critically ill host.

In critically ill, mechanically ventilated patients, the oral cavity serves as a reservoir for pathogenic microorganisms that can colonize the lower respiratory tract. Chlorhexidine 0.12%, through disruption of bacterial cell membranes and its well‐documented substantivity, provides sustained antimicrobial activity and effectively reduces microbial load and biofilm maturation.

In this highly vulnerable population, inadequate oral hygiene is not a benign omission; rather, it contributes to systemic inflammatory amplification, increased risk of nosocomial infection, and overall morbidity. For patients with mitochondrial instability and limited physiological reserve, even modest infectious or inflammatory insults may precipitate clinical deterioration. Accordingly, the structured oral care protocol instituted in this case, comprising antiseptic biofilm control, meticulous mucosal cleansing, and maintenance of tissue hydration, should be understood not merely as a preventive adjunct but as an integral therapeutic intervention. By reducing oral microbial burden and inflammatory signaling, this approach aligns local oral management with broader systemic risk mitigation, reinforcing dentistry's role as a critical component of comprehensive ICU care [15].

This intrinsic metabolic vulnerability may also modify tissue responses to prolonged pharmacologic exposure, providing a biologically plausible explanation for the severe DIGO observed in the present patient under long‐term anticonvulsant and immunosuppressive therapy [6].

Electrosurgical approaches, as outlined by Tungare and Paranjpe (2021) [7], offer recognized advantages, including superior intraoperative hemostasis, enhanced visualization of the surgical field, and reduced operative time, considerations of particular relevance in medically fragile patients. However, anesthetic management in individuals with mitochondrial disorders requires heightened vigilance, as metabolic stress, impaired oxidative phosphorylation, and altered energy homeostasis may precipitate perioperative decompensation [4, 14].

A crucial aspect of this case was the prolonged absence of dental follow‐up: the patient had remained without professional oral care for approximately four years before referral to our tertiary institution, where routine dental evaluation was finally established. This extended period without surveillance likely contributed substantially to the severity and progression of the gingival overgrowth in the context of chronic polypharmacy and critical illness.

Comprehensive multidisciplinary discussion proved fundamental in guiding clinical decision‐making. The deliberation focused particularly on balancing the theoretical infectious benefits of surgical tissue reduction against the significant risks of postoperative complications, including impaired wound healing in an immunosuppressed patient and the potential for systemic destabilization. The possibility of reducing anticonvulsant and immunosuppressive therapy was carefully evaluated; however, given the imperative need for seizure control and graft preservation, modification of phenytoin, sodium valproate, or tacrolimus was deemed clinically inappropriate. Likewise, despite its theoretical local benefits, gingivoplasty was ultimately considered disproportionate to the systemic risks in this specific context.

Accordingly, the team collectively determined that conservative management with close clinical monitoring represented the most ethically and medically sound course of action. This decision underscores the necessity of meticulous risk stratification, multidisciplinary coordination, and the proportionate application of care when managing complex mitochondrial disease in critically ill pediatric patients.

The early integration of palliative care principles in severe mitochondrial disorders such as AHS is not optional but imperative, given the substantial symptomatic burden and the relentless, irreversible progression of neurological and hepatic dysfunction. In these patients, care must extend beyond life‐prolonging interventions to encompass meticulous symptom control and preservation of dignity and comfort. The literature consistently demonstrates that individuals receiving palliative care exhibit a high prevalence of treatable oral conditions, including xerostomia, oral candidiasis, dysphagia, mucositis, and orofacial pain, which exert a profound impact on nutrition, communication, susceptibility to infection, and overall quality of life [2, 7, 16]. Despite their frequency and clinical relevance, such oral manifestations are frequently underrecognized in critically ill populations.

Within this framework, the integration of dental professionals into the multidisciplinary team alongside neurologists, hepatologists, intensivists, and transplant specialists is particularly significant [1, 16, 17, 18]. In medically complex patients undergoing prolonged ICU hospitalization and polypharmacy, structured dental oversight contributes not only to local symptom mitigation but also to systemic risk reduction through infection control and modulation of the inflammatory burden. In the present case, chronic exposure to anticonvulsants and post‐transplant immunosuppressants further increased vulnerability to adverse oral events, including severe DIGO, underscoring the need for systematic monitoring and protocol‐driven oral care [7, 8, 17, 18].

By documenting the convergence of mitochondrial disease, liver transplantation, chronic intensive care dependence, and pronounced drug‐induced gingival hypertrophy, this report expands the current literature. It reinforces a critical paradigm: structured dental integration is not ancillary but foundational in the comprehensive management of rare metabolic disorders with high systemic complexity.

## 4 Conclusion

The early institution of structured oral hygiene protocols, vigilant clinical monitoring, and judicious surgical decision‐making guided by rigorous risk‐benefit analysis is essential to mitigate infectious risk and preserve quality of life in this population. To our knowledge, this is the first report documenting an association between AHS and severe DIGO. This case underscores the imperative of proactive dental surveillance and integrated oral care strategies for patients with rare mitochondrial disorders.

## Author Contributions

Marcus Vinícius Bueno is involved in writing – review and editing, Writing – original draft, and supervision. Renata Zoraida Rizental Delgado and Karem Ortega are involved in review, and editing. Juliana Bertoldi Franco is involved in conceptualization, methodology, and writing.

## Funding

This research did not receive any specific funding from public, commercial, or non‐profit funding agencies.

## Ethics Statement

All Authors declare that no ethical statement will be required for this manuscript.

## Conflicts of Interest

The authors declare no conflicts of interest.
